# Multiplex Antibody Detection for Noninvasive Genus-Level Diagnosis of Prosthetic Joint Infection

**DOI:** 10.1128/JCM.02885-15

**Published:** 2016-03-25

**Authors:** Simon Marmor, Thomas Bauer, Nicole Desplaces, Beate Heym, Anne-Laure Roux, Olivier Sol, Julie Rogé, Florence Mahé, Laurent Désiré, Philippe Aegerter, Idir Ghout, Jacques Ropers, Jean-Louis Gaillard, Martin Rottman

**Affiliations:** a Service de Chirurgie Orthopédique, Groupe Hospitalier Diaconesses Croix Saint-Simon, Paris, France; b Service de Chirurgie Orthopédique et Traumatologie, Hôpital Ambroise Paré (Assistance Publique–Hôpitaux de Paris [AP-HP]), Boulogne-Billancourt, France; c Service de Microbiologie, Groupe Hospitalier Diaconesses Croix Saint-Simon, Paris, France; d Laboratoire de Microbiologie, Hôpital Ambroise Paré (AP-HP), Boulogne-Billancourt, France; e UMR 1173, UFR Simone Veil, Université de Versailles Saint-Quentin-en-Yvelines, Montigny-le-Bretonneux, France; f DIAXONHIT, Paris, France; g Unité de Recherche Clinique Paris Île-de-France Ouest, Hôpital Ambroise Paré (AP-HP), Boulogne-Billancourt, France; h Laboratoire de Microbiologie, Hôpital Raymond Poincaré (AP-HP), Garches, France

## Abstract

We developed and evaluated a multiplex antibody detection-based immunoassay for the diagnosis of prosthetic joint infections (PJIs). Sixteen protein antigens from three Staphylococcus species (Staphylococcus aureus, Staphylococcus epidermidis, and Staphylococcus lugdunensis) (8 antigens), Streptococcus agalactiae (4 antigens), and Propionibacterium acnes (4 antigens) were selected by comparative immunoproteomics using serum samples from PJI cases versus controls. A bead-based multiplex immunoassay that measured serum IgG against purified, recombinant forms of each of the 16 antigens was developed. We conducted a prospective study to evaluate the performance of the assay. A PJI was defined by the presence of a sinus tract and/or positive intraoperative sample cultures (at least one sample yielding a virulent organism or at least two samples yielding the same organism). A total of 455 consecutive patients undergoing revision or resection arthroplasty (hip, 66.3%; knee, 29.7%; shoulder, 4%) at two French reference centers for the management of PJI were included: 176 patients (38.7%) were infected and 279 (61.3%) were not. About 60% of the infections involved at least one of the species targeted by the assay. The sensitivity/specificity values were 72.3%/80.7% for targeted staphylococci, 75%/92.6% for S. agalactiae, and 38.5%/84.8% for P. acnes. The assay was more sensitive for infections occurring >3 months after arthroplasty and for patients with an elevated C-reactive protein (CRP) or erythrocyte sedimentation rate (ESR). However, it detected 64.3% and 58.3% of targeted staphylococcal infections associated with normal CRP and ESR values, respectively. This new multiplex immunoassay approach is a novel noninvasive tool to evaluate patients suspected of having PJIs and provides information complementary to that from inflammatory marker values.

## INTRODUCTION

Joint replacements are among the most successful surgical procedures with the number of procedures performed in 2010 exceeding 1 million (1) and expected to exceed 4 million yearly by 2030 (2). Prosthetic joint infection (PJI) is a major complication associated with increased morbidity, poor functional outcome, and increased use of health care resources (3, 4). It occurs in up to 2.4% of primary arthroplasties and in 10 to 25% of revision arthroplasties (5, 6). Staphylococcus aureus and coagulase-negative staphylococci are the most frequent organisms involved, followed by streptococci-enterococci, aerobic Gram-negative bacilli, Propionibacterium acnes, and other anaerobes (7–9).

The distinction between PJI and mechanical prosthetic dysfunction preoperatively is often difficult (8) but is essential because the two diagnoses require radically different surgical strategies. The current diagnostic algorithms involve serological screening with the erythrocyte sedimentation rate (ESR) and C-reactive protein (CRP), followed by joint aspiration if either is elevated (10). However, the ESR and CRP are both markers of inflammation and do not provide information on the infecting agent. Furthermore, their values in PJI may be normal (10, 11), delaying the management of patients with infections and triggering costly investigations.

Advances in multiplexing technology have paved the way for *in vitro* diagnostic tests that quantify antibodies to multiple antigens in a single reaction (12). However, this approach has rarely been applied to bacterial infectious diseases (13, 14) and never to PJIs. The serological diagnosis of systemic staphylococcal infection has been previously attempted with as many as seven antigenic preparations evaluated in combination (15) but with no subsequent use of the assay, and recent evaluations have confirmed the lack of clinical benefit of the commercially available antistaphylolysin and antinuclease assays (16).

We developed a multiplex antibody detection-based immunoassay using a panel of recombinant antigens from major PJI pathogens (Staphylococcus spp.,Streptococcus agalactiae, and Propionibacterium acnes) that provides independent results for each of the targeted pathogens. We performed a prospective study to evaluate its performance in two French orthopedic centers specializing in the management of complex bone and joint infections.

(This work was presented in part at the 115th General Meeting of the American Society for Microbiology, New Orleans, LA, 30 May to 2 June 2015, and at the 25th European Congress of Clinical Microbiology and Infectious Diseases, Copenhagen, Denmark, 25 to 28 April 2015.)

## MATERIALS AND METHODS

### Study design and oversight.

This was a prospective, multicenter, noninterventional study performed in patients scheduled for revision or resection arthroplasty for a knee, hip, or shoulder implant. The objective of the study was to assess the performance of the multiplex immunoassay, using as reference the microbiological cultures performed on periprosthetic tissue samples obtained during surgery.

The protocol and information sheet were approved by the institutional review board (IRB) CPP Île-de-France XI. The database was authorized by the Commission Nationale Informatique Libertés (French privacy watchdog), and all patients were informed before inclusion and given the opportunity to opt out. The study was performed in accordance with the principles of the Declaration of Helsinki and good clinical practice guidelines.

### Study population and procedures.

We included all consecutive adult patients with total hip, knee, or shoulder prosthesis who underwent revision or resection arthroplasty between 25 June 2012 and 23 June 2014 at two French reference centers for the management of bone and joint infections. The main exclusion criteria were joint revision surgery for more than one implant, HIV infection, and cancer chemotherapy.

Blood samples for the multiplex immunoassay were taken at the inclusion visit. Serum aliquots were frozen and stored at −20°C. All samples were blinded with respect to all patient information before processing. The multiplex immunoassay and microbiological cultures using intraoperative tissue samples were performed for all patients, regardless of clinical suspicion of PJI. Antibiotics were withheld at least 2 weeks prior to collection of intraoperative tissue samples.

### Selection of antigens included in the multiplex immunoassay.

**FIG 1:**
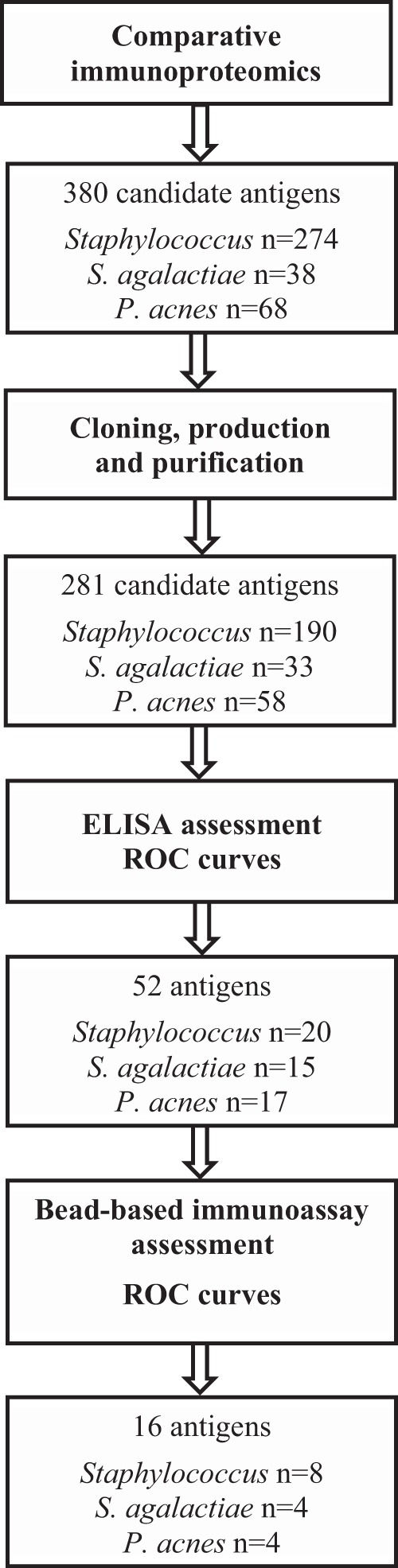
Selection of antigens included in the multiplex immunoassay.

Antigens were selected by comparative immunoproteomics followed by enzyme-linked immunosorbent assay (ELISA) and Luminex assessments (Fig. 1). Serum samples were from PJI cases involving a single species (S. aureus, 37; Staphylococcus epidermidis, 45; Staphylococcus lugdunensis, 4; Streptococcus agalactiae, 13; P. acnes, 31); controls included healthy blood donors (*n* = 98) and patients (*n* = 66) who underwent bone and joint replacement surgery at least 2 years previously without any abnormal signs or symptoms at follow-up (“orthopedic controls”). Strains of the targeted species were S. aureus Mu50, COL, and USA 300, S. epidermidis RP62A (ATCC 35894) and ATCC 12228, S. lugdunensis CIP 103642, S. agalactiae CIP 82.45, and P. acnes KPA171202 (type IB).

A total of 380 candidate antigens (staphylococci, 274; S. agalactiae, 38; P. acnes, 68) were identified by comparative immunoproteomics using pooled serum samples from PJI cases and controls collected during the course of a separate multicentric prospective study to develop the assay (for further details, see the supplemental material). Among them, 281 antigens (staphylococci, 190; S. agalactiae, 33; P. acnes, 58) were successfully expressed in Escherichia coli. The antigens were produced as fusion proteins with a His_6_ tag at their N-terminal end; they were purified with the Äkta Xpress chromatography system (GE Healthcare, Velizy-Villacoublay, France) at a minimum purity of 95%.

A first screening of the purified recombinant antigens was performed using an ELISA. The purified antigens were coated onto the wells of Nunc MaxiSorp microtiter plates (Thermo Fisher Scientific, MA, USA). Serum IgG binding was detected with a goat anti-human IgG horseradish peroxidase-conjugated antibody. The optical density at 450 nm (OD_450_) values were analyzed with TANAGRA software, version 1.4.31 (http://eric.univ-lyon2.fr/∼ricco/tanagra/en/tanagra.html). The ability of each candidate antigen to discriminate PJI cases from controls was evaluated using receiver operator characteristic (ROC) curves, allowing the identification of 52 relevant antigens (staphylococci, 20; S. agalactiae, 15; P. acnes, 17).

The 52 antigens identified by the ELISA were further assessed using the Luminex technology. Purified recombinant proteins (50 μg/ml) were coupled to Magplex beads (Luminex, Austin, TX, USA). Serum IgG binding was detected with R-phycoerythrin-conjugated AffiniPure goat anti-human IgG (Moss, Inc., Pasadena, MD). Median fluorescence intensity (MFI) values were determined using a Magpix instrument and xPONENT software. ROC curves were used to select the 16 final proteins included in the immunoassay: 8 staphylococcal proteins (3 proteins involved in biofilm formation, 3 lipoproteins, 1 virulence factor, 1 putative adherence factor), 4 S. agalactiae proteins (1 virulence factor, 1 protein putatively involved in metabolism, 2 proteins with unknown function), and 4 P. acnes proteins (2 membrane proteins with transport functions, 1 protein involved in metabolism, 1 putative oxidoreductase).

### Multiplex immunoassay.

The immunoassay (research use only version of BJI InoPlex; Diaxonhit, Paris, France) is a bead-based multiplex assay (Magplex beads) designed to target three Staphylococcus species (S. aureus, S. epidermidis, and S. lugdunensis), Streptococcus agalactiae, and P. acnes. It quantifies the patient's IgG binding to the purified, recombinant forms of 16 proteins (see above). The serum samples were diluted 1:70. IgG binding was detected with R-phycoerythrin-conjugated goat anti-human IgG. Positive-control, negative-control, and calibrating sera were included in each series. Measurements were performed on a Magpix instrument. MFI values were processed using proprietary software (Diaxonhit). Results are expressed as positive, negative, or undetermined for staphylococci, S. agalactiae, and P. acnes.

### Bacteriological methods.

At least three intraoperative periprosthetic tissue samples or small hardware obtained using a sterile instrument set were placed in sterile doubly wrapped 30-ml containers and processed within 2 h as previously described (17). Briefly, samples were mechanically disrupted in sterile water with stainless steel beads using a Retsch MM 400 bead mill (Verder, Cergy-Pontoise, France). Bead-milled sample suspensions were then cultured on solid medium (Columbia sheep blood agar incubated under aerobic and anaerobic atmospheres or chocolate agar under 5% CO_2_) and on liquid aerobic (brain heart infusion) and anaerobic (Schaedler's) broths terminally subcultured after 15 days. Enumeration and differential counting were performed on intraoperative synovial aspirations, and an identical culture scheme was applied to these samples. All manipulations were performed using a safety cabinet. Isolates were identified by mass spectrometry using a Microflex LT instrument and the current CE-marked IVD Biotyper software (Bruker Daltonique, Wissenbourg, France).

### Definition of prosthetic joint infection.

An infection was defined by (i) the presence of a sinus tract and/or (ii) at least one intraoperative sample positive in culture with a virulent organism or at least two intraoperative samples positive in culture with the same microorganism (same species and same susceptibility profile). The absence of infection was defined as no sinus tract and no positive culture for any sample or a single culture positive for a nonvirulent organism. An infection was considered polymicrobial when multiple organisms fulfilled the microbiological criteria for infection. This definition takes into account the major infection criteria of the Infectious Diseases Society of America (IDSA) (10) and the Musculoskeletal Infection Society (MSIS) (18, 19) guidelines. Of the supportive criteria, only single isolates of virulent microorganisms have been considered due to incomplete data sets.

### Study endpoints.

The sensitivity for each microorganism targeted by the immunoassay was defined as the proportion of patients found seropositive for this microorganism among those infected with the same microorganism. The specificity for each targeted microorganism was defined as the proportion of patients seronegative for this microorganism among noninfected patients.

### Statistical analysis.

The sensitivity and specificity of the immunoassay were estimated along with their two-sided 95% Clopper-Pearson exact confidence intervals for binomial proportions. The categorical and continuous variables were compared using Fisher's exact test, Student's *t* test, and the Wilcoxon rank sum test as appropriate. Two-sided *P* values of <0.05 were considered statistically significant. All calculations were performed using R version 2.13 (20).

## RESULTS

### Study population.

**FIG 2:**
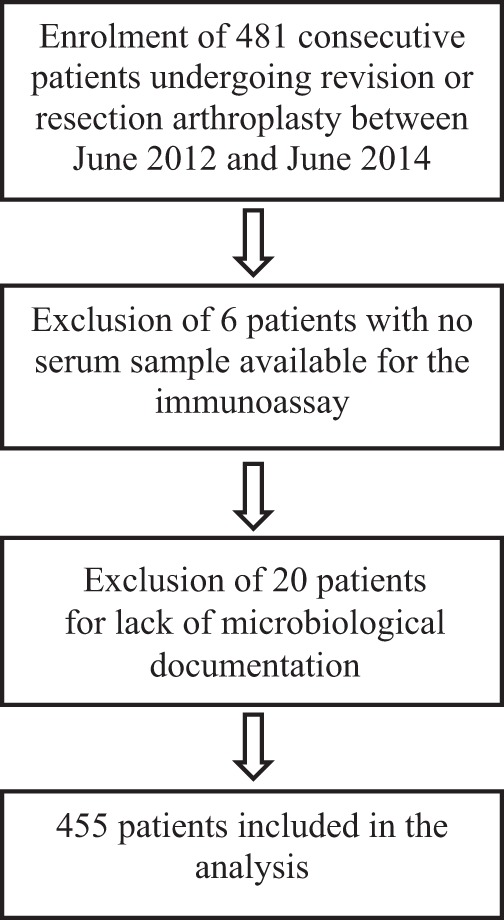
Flowchart of patients' inclusion.

A total of 481 consecutive patients were enrolled in the study, and 455 were included in the analysis (Fig. 2); 279 (61.3%) were defined as noninfected and 176 (38.7%) were defined as infected. The characteristics of the infected and noninfected patients were significantly different except for age, excess weight, and the proportion undergoing a first prosthesis replacement (Table 1). In particular, the ratio of men to women was higher among infected than noninfected patients, and infected patients were more likely to have had a prosthesis inserted in the past 3 months (Table 1).

**TABLE 1:** Baseline characteristics of the population studied

Characteristic^*a*^	Total (*n* = 455)	Noninfected (*n* = 279)	Infected (*n* = 176)	*P*^*b*^
Age (mean ±SD) (yr)	69.6 ± 12.1	70.5 ± 11	68.3 ± 13.6	0.083
Female (no./total no. [%])	237/453 (52.3)	162/277 (58.5)	75/176 (42.6)	0.001
Wt (median [IQR]) (kg)	75 (64 to 87)	74 (62 to 85)	79.5 (65.8 to 92.2)	0.003
Excess wt (median [IQR]) (kg)	5.7 −3.6 to 15.8	5.2 −3.7 to 14.3	6.4 −3.2 to 19.1	0.077
First implant revision (no./total no. [%])	264/409 (64.5)	172/257 (66.9)	92/152 (60.5)	0.23
Site of prosthesis (no./total no. [%])				
Hip	301/454 (66.3)	202/278 (72.7)	99/176 (56.2)	<0.001
Knee	135/454 (29.7)	71/278 (25.5)	64/176 (36.4)	
Shoulder	18/454 (4)	5/278 (1.8)	13/176 (7.4)	
Time since prosthesis insertion (no./total no. [%])				
≤3 mo	37/441 (8.4)	7/272 (2.6)	30/169 (17.8)	<0.001
>3 mo	404/441 (91.6)	265/272 (97.4)	139/169 (82.2)	
Sinus tract (no./total no. [%])	46/455 (10.1)	-	46/176 (26.1)	
CRP of ≥10 mg/liter^*c*^	217/442 (49.1)	71/268 (26.5)	146/174 (83.9)	<0.001
ESR of ≥30 mm/h^*c*^	169/389 (41.6)	50/250 (20)	112/139 (80.6)	<0.001

a IQR, interquartile range; CRP, serum C-reactive protein; ESR, erythrocyte sedimentation rate.

b Infected *versus* non-infected cases.

c The cutoffs are taken from reference 8.

### Organisms recovered from intraoperative tissue cultures.

The species most frequently cultured from infected cases (Table 2) were S. aureus (33% of infections), S. epidermidis (22.2%), P. acnes, and Enterococcus faecalis (7.4% each); S. agalactiae was involved in 4.5% of infections and was the most frequent streptococcal species. S. aureus (54.2%) and S. epidermidis (37.5%) were also the most frequent species recovered from polymicrobial infections, which accounted for 14.4% (24/167) of the culture-positive cases (see Table S1 in the supplemental material). Overall, 59.2% (100/169) of the culture-positive infections involved at least one of the organisms targeted by the immunoassay.

**TABLE 2:** Microbial species involved in the infected cases^*a*^

Microbial species	All sites (*n* = 176)^*b*^	Hip (*n* = 99)	Knee (*n* = 64)	Shoulder (*n* = 13)
Staphylococcus species				
S. aureus	58 (33)	33 (33.3)	22 (34.4)	3 (23.1)
S. epidermidis	39 (22.2)	22 (22.2)	14 (21.9)	3 (23.1)
S. lugdunensis	9 (5.1)	3 (3)	6 (9.4)	0
S. capitis	7 (4)	5 (5)	2 (3.1)	0
S. caprae	1 (0.6)	1 (1)	0	0
S. haemolyticus	1 (0.6)	0	1 (1.6)	0
S. hominis	1 (0.6)	1 (1)	0	0
S. xylosus	1 (0.6)	0	1 (1.6)	0
Staphylococcus sp.	0	0	0	0
Streptococcus species				
S. agalactiae	8 (4.5)	3 (3)	4 (6.2)	1 (8)
S. bovis	1 (0.6)	0	1	0
S. dysgalactiae	1 (0.6)	1 (1)	0	0
S. oralis	2 (1.1)	0	2 (3.1)	0
S. pneumoniae	2 (1.1)	1	0	1 (8)
S. sanguinis	1 (0.6)	1 (1)	0	0
Propionibacterium species				
P. acnes	13 (7.4)	6 (6.1)	2 (3.1)	5 (38.5)^*c*^
P. avidum	3 (1.7)	2 (2)	0	1 (8)
Other bacterial species				
Acinetobacter ursingii	1 (0.6)	0	1 (1.6)	0
Anaerococcus hydrogenalis	1 (0.6)	0	1 (1.6)	0
Corynebacterium striatum	3 (1.7)	2 (2)	1 (1.6)	0
Enterobacter cloacae	8 (4.5)	6 (6.1)	2 (3.1)	0
Enterococcus faecalis	13 (7.4)	10 (10.1)	2 (3.1)	1 (8)
Enterococcus faecium	2 (1.1)	1 (1)	1 (1.6)	0
Escherichia coli	6 (3.4)	6 (6.1)	0	0
Finegoldia magna	4 (2.3)	2 (2)	1 (1.6)	1 (8)
Granulicatella adjacens	1 (0.6)	1 (1)	0	0
Klebsiella pneumoniae	2 (1.1)	2 (2)	0	0
Morganella morganii	1 (0.6)	1 (1)	0	0
Peptoniphilus harei	2 (1.1)	1 (1)	1 (1.6)	0
Pseudomonas aeruginosa	5 (2.8)	2 (2)	3 (4.7)	0
Salmonella sp.	2 (1.1)	2 (1)	0	0
Fungal species				
Candida albicans	1 (0.6)	0	1 (1.6)	0

a Microbial species recovered from at least one intraoperative sample (virulent organisms) or from at least two intraoperative samples (nonvirulent organisms). Data are number (%) of cases; the percentage represents the proportion of all infected cases, culture-proven or not.

b Nine infections with sinus tracts were associated with negative (*n* = 7) or nonsignificant (*n* = 2) microbiological cultures.

c P. acnes was found significantly more frequently in the shoulder than in the hip (*P* = 0.0001) or knee (*P* = 0.0002).

The global distribution of species was similar for hip and knee infections (Table 2). However, the most frequent species after S. aureus and S. epidermidis were E. faecalis, P. acnes, Enterobacter cloacae, and E. coli in the hip and S. lugdunensis and S. agalactiae in the knee. P. acnes was significantly more frequent in shoulder infections than in hip (*P* = 0.0001) or knee (*P* = 0.0002) infections (Table 2).

P. acnes (20/279, 7.2%) and S. epidermidis (14/279, 5%) were the most frequent species recovered in single positive cultures from noninfected cases.

### Performance of the immunoassay.

The sensitivity/specificity values for the immunoassay were calculated by excluding undetermined results and were 72.3%/80.7% (95% confidence intervals, 62.7 to 80.7/75.6 to 85.1) for staphylococci, 75%/92.6% (38.8 to 95.6/89 to 95.3) for S. agalactiae, and 38.5%/84.8% (15.7 to 65.9/80.2 to 88.7) for P. acnes (Table 3). The values calculated by classifying undetermined results as either positive or negative are presented in Table S2 in the supplemental material.

**TABLE 3:** Performance of the multiplex immunoassay^*a*^

Organism(s)	All cases		Site of prosthesis				Time since implantation			
			Hip		Knee		≤3 mo		>3 mo	
	Sensitivity	Specificity	Sensitivity	Specificity	Sensitivity	Specificity	Sensitivity	Specificity	Sensitivity	Specificity
Staphylococci targeted	68/94 (72.3) [62.7–80.7]	213/264 (80.7) [75.6–85.1]	38/52 (73.1) [59.9–83.8]	159/190 (83.7) [77.9–88.4]	29/39 (74.4) [59–86.2]	50/68 (73.5) [62.1–83]	9/15 (60) [34.5–81.9]	3/7 (42.9) [12.3–78.4]	57/75 (76) [65.4–84.6]	205/250 (82) [76.9–86.4]
S. aureus	36/54 (66.7) [53.4–78.2]		21/30 (70) [52–84.3]		14/21 (66.7) [44.9–84.1]		7/11 (63.6) [33.6–87.2]		27/39 (69.2) [53.6–82.1]	
S. epidermidis	26/35 (74.3) [58–86.7]		16/21 (76.2) [54.9–90.7]		10/13 (76.9) [49.1–93.8]		2/4 (50) [9.4–90.6]		24/31 (77.4) [60.4–89.6]	
S. lugdunensis	9/9 (100) [71.7–100]		3/3 (100) [36.8–100]		6/6 (100) [60.7–100]				9/9 (100) [71.7–100]	
S. agalactiae	6/8 (75) [38.8–95.6]	250/270 (92.6) [89–95.3]	3/3 (100) [36.8–100]	181/195 (92.8) [88.5–95.9]	2/4 (50) [9.4–90.6]	63/69 (91.3) [82.8–96.4]	0/1 (0) [0–95]	7/7 (100) [65.2–100]	6/7 (85.7) [47–99.3]	237/256 (92.6) [88.9–95.3]
P. acnes	5/13 (38.5) [15.7–65.9]	235/277 (84.8) [80.2–88.7]	1/6 (16.7) [0.8–59.1]	172/200 (86) [80.7–89.5]	1/2 (50) [2.5–97.5]	60/71 (84.5) [74.7–91.6]		7/7 (100) [65.2–100]	4/12 (33.3) [11.6–62.3]	222/263 (84.4) [79.6–88.4]

a Data are no. (%) [95% confidence interval].

The sensitivity values were not significantly lower for S. aureus than for S. epidermidis infections (66.7% [95% confidence interval, 53.4 to 78.2] versus 74.3% [58 to 86.7]; *P* = 0.49); the immunoassay, however, detected 73.8% (31/42) and 85.7% (12/14) of S. aureus infections associated with two or more positive samples or presenting with a sinus tract, respectively (Table 3). All S. lugdunensis infections (9/9) were detected. The immunoassay tended to be more sensitive for staphylococcal infections occurring >3 months after arthroplasty (76% [65.4 to 84.6] versus 60% [34.5 to 81.9]; *P* = 0.214) (Table 3). The performance of the immunoassay was similar for the hip and knee (Table 3). Although the total number of shoulder infections was small (*n* = 13), the immunoassay was able to detect the single S. agalactiae case and three of the five P. acnes cases.

The immunoassay results were found to be “false” positive in 54.5% (6/11) of infections involving staphylococcal species other than S. aureus, S. epidermidis, or S. lugdunensis, 22.7% (5/22) of infections involving streptococci other than S. agalactiae and two of the three infections involving Propionibacterium avidum. The results were positive in 5 of the 13 cases with a single positive S. epidermidis culture (38.5%) and in only 2 of the 21 cases with a single positive P. acnes culture (9.5%).

### Relationship with ESR and CRP.

**FIG 3:**
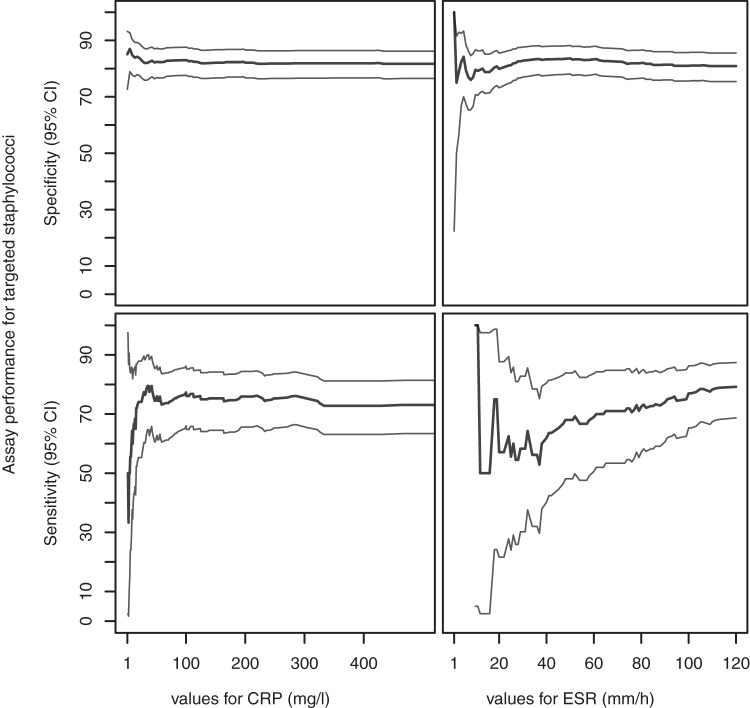
Immunoassay sensitivity and specificity with respect to targeted staphylococci for all values of CRP and ESR. The plots show the sensitivities and specificities of the assay (versus targeted staphylococci) according to the values of CRP and ESR. The inner bold lines are estimated sensitivity and specificity values; the outer lines are pointwise 95% confidence bands (CI).

The immunoassay was more sensitive when the ESR or CRP was elevated, with unchanged specificity. The largest increases were observed with the ESR for staphylococcal species not included in the immunoassay (85.7% [95% confidence interval, 47 to 99.3] for an elevated ESR versus 0% [0 to 52.7] for a normal ESR; *P* = 0.0152) and S. epidermidis (90.9% [73.1 to 98.4] for an elevated ESR versus 50% [14.7 to 85.3] for a normal ESR; *P* = 0.05) (Table 4). Moreover, the ESR was directly correlated with the sensitivity of the immunoassay for staphylococci, but this was not the case for CRP (Fig. 3).

**TABLE 4:** Performance of the multiplex immunoassay in relation to ESR and CRP results^*a*^

Organism(s)	ESR elevated^*b*^		ESR normal		CRP elevated^*c*^		CRP normal	
	Sensitivity	Specificity	Sensitivity	Specificity	Sensitivity	Specificity	Sensitivity	Specificity
Staphylococci targeted	50/60 (83.3) [72.3–91.2]	33/45 (73.3) [59.1–84.7]	7/12 (58.3) [30.2–82.8]	157/190 (82.6) [76.7–87.5]	59/79 (74.7) [64.2–83.3]	54/68 (79.4) [68.6–87.8]	9/14 (64.3) [37.6–85.6]	152/185 (82.2) [76.1–87.2]
S. aureus	26/34 (76.5) [60.2–88.4]	—^*d*^	3/6 (50) [14.7–85.3]	—	34/48 (70.8) [56.9–82.3]	—	2/5 (40) [7.3–81.8]	—
S. epidermidis	20/22 (90.9) [73.1–98.4]	—	3/6 (50) [14.7–85.3]	—	21/27 (77.8) [59.4–90.5]	—	5/8 (62.5) [27.8–89.4]	—
S. lugdunensis	6/6 (100) [60.7–100]	—	1/1 (100) [5–100]	—	7/7 (100) [65.2–100]	—	2/2 (100) [22.4–100]	—
Other staphylococci	6/7 (85.7) [47–99.3]	—	0/4 (0) [0–52.7]	—	5/9 (55.6) [24–84]	—	1/2 (50) [2.5,97.5]	—
S. agalactiae	6/8 (75) [38.8–95.6]	43/49 (87.8) [76.3–94.9]	—	180/192 (93.8) [89.6–96.6]	4/5 (80) [33.4–99]	64/70 (91.4) [83–96.5]	1/2 (50) [2.5–97.5]	176/189 (93.1) [88.8–96.1]
P. acnes	3/6 (50) [14.7–85.3]	45/50 (90) [79.2–96.2]	0/4 (0) [0–52.7]	166/198 (83.8) [78.2–88.5]	4/8 (50) [18.4–81.6]	59/71 (83.1) [73–90.5]	1/5 (20) [1–66.6]	167/195 (85.6) [80.2–90.1]

a Data are no. (%) [95% confidence interval].

b Levels of ≥30 mm/h.

c Levels of ≥10 mg/liter.

d —, not applicable.

However, the immunoassay was also able to identify infections associated with normal levels of inflammatory markers (Table 4). It was positive in 64.3% and 58.3% of targeted staphylococcal infections associated with normal levels of CRP or ESR, respectively, and was also positive in one of the two S. agalactiae infections with a normal CRP.

## DISCUSSION

This study provides evidence that the noninvasive detection of serum antibodies can indirectly diagnose PJI. Different carbohydrate antigen preparations of S. epidermidis have been considered in this indication, but the assays have not been commercialized or evaluated beyond preliminary studies (21, 22). The antigens included in the assay were all identified by comparative immunoproteomics, i.e., by comparing the antibody response profile of arthroplasty patients with PJIs to those without infection. The identification of bacterial targets of humoral immunity in PJI patients allowed us to select the antigens most relevant to PJI, in which pathogens commonly undergo phenotypic changes (23–25). All antigens included in our assay were synthesized as recombinant proteins, and some were further engineered to improve their solubility and stability or to enhance the specificity of the response through the deletion of nonrelevant epitopes.

Our main objective was to detect PJIs caused by staphylococci, which cause more than 50% of PJIs and are among the most deleterious and antibiotic-resistant agents (8). Antigens derived from S. aureus, S. epidermidis, and S. lugdunensis, the three most prevalent staphylococcal species (7, 8), were included in the assay. The sensitivity/specificity values of the assay for the three species were overall 72.3%/80.7%; the sensitivity was nonsignificantly lower for S. aureus, due to the weaker ability of the assay to detect cases with a single positive culture. The sensitivity/specificity values reached 76%/82% when the assay was performed ≥3 months after surgery (>80% of targeted staphylococcal infections). This performance is in the range of that reported with the ESR and CRP (26, 27), with the immunoassay showing a slightly lower sensitivity than either marker in our patients. However, the immunoassay detected around 60% of targeted staphylococcal infections associated with a normal ESR or CRP, demonstrating its ability to identify infection in the absence of systemic inflammation and to identifying patients otherwise overlooked by most diagnostic algorithms (10, 11, 18). The assay therefore improves the specificity of the currently available rule-out assays.

The limited number of S. agalactiae PJI cases prevented us from definitively assessing the performance of the immunoassay for this pathogen, the most common streptococcal species causing PJIs (7, 28). However, the overall performance was similar to that found with targeted staphylococci and even better in cases occurring >3 months after arthroplasty (sensitivity/specificity, 85.7%/92.6%), which account for the vast majority of S. agalactiae PJIs (29). The sensitivity of the immunoassay for P. acnes was much lower at <40% when all PJIs were considered and 50% for PJIs in patients with elevated CRP or ESR. The antigens included in the assay may not be optimal. Bacteria were grown in liquid medium and not in biofilm, and the immunoproteomic evaluation might have not identified the relevant antigens solely expressed in sessile bacteria. The antigens were derived from an IB strain and might be less sensitive with other P. acnes types frequently isolated in PJIs such as type II (30). The low sensitivity of our assay may also reflect the low virulence of P. acnes. Indeed this organism is often isolated in cases of fortuitous intraoperative cultures (7, 31–34), and there are instances of clinically silent P. acnes PJIs defined bacteriologically and amenable to limited therapy (33). The correlation between serologically silent P. acnes and PJI diagnosed by fortuitous intraoperative culture should be explored. To better understand the relevance of the immunoassay, it would also be interesting to focus on shoulder arthroplasty, where the prevalence of P. acnes is highest (35, 36).

We cannot rule out a bias resulting from the recruitment of patients from infection reference centers, which guaranteed a large number of PJI cases and optimal bacteriological procedures. This may have resulted in an overrepresentation of the more challenging cases and may detract from the everyday use of the assay. Our data should therefore be validated by studies conducted in primary care settings, where the immunoassay would be used by practitioners less often confronted with PJIs, in conjunction with the ESR and CRP. These studies will allow determination of the positive and negative predictive values of the immunoassay, an analysis that would have been irrelevant in our study due to the abnormally high prevalence of PJIs.

Several culture methods have been proposed for the microbiological documentation of PJIs. The bead-milling of multiple intraoperative samples was routinely performed at both centers when the study was designed (17). This technique was subsequently evaluated independently and recognized in the literature (10). It mechanizes the process previously used to define the interpretation criteria of multiple intraoperative cultures (37). Implant sonication (38), which was not used routinely in either participating center, was not performed in the patients enrolled. The molecular detection of targeted organisms might have been an interesting comparator for discrepant results between culture and serology. However, 16S PCR and sequencing on bead-milled periprosthetic tissues has shown limited benefit in patients without previous antimicrobial treatment (39), and validated species-specific PCR protocols, including those for Staphylococcus spp., S. agalactiae, and P. acnes, were not available at the time of the study design. Molecular techniques would likely improve the etiologic documentation of cases not fulfilling the bacteriological diagnostic criteria, and future studies might benefit from their use. These techniques might also provide evidence of occult polymicrobial infection involving fastidious organisms such as P. acnes, likely to be overgrown by more hardy pathogens.

The immunoassay also has limitations. First, bacterial species not covered by the assay account for 40% of total PJIs. The assay sensitivity and specificity in diagnosing PJIs caused by any etiological agent, therefore, only reach 67.7% and 65.6%, respectively. However, the assay was developed to provide a noninvasive genus-level documentation of infection with selected microorganisms, in conjunction with the other tests used to establish infection; moreover, the multiplex nature of the assay allows for the subsequent broadening of the bacterial species included. Second, the sole detection of IgGs does not provide information on the time frame of the infection and may yield false-negative results when performed too soon after the onset of infection, as suggested by the lower performance of the assay within the first 3 months following the arthroplasty. Third, cross-reactivity between antigens from species belonging to the same genus (*i.e*., Staphylococcus) prevents species-level identification of the causative agent; however, it allows the diagnosis of PJIs caused by closely related species of the same genus. Further studies will allow us to determine the specificity of the antibody response observed in PJI cases involving nontargeted staphylococci, streptococci, and propionibacteria and might lead to broadening of the range of identified species. Finally, “real-life” patients with sera yielding undetermined results (6% of serum samples from PJI cases due to the targeted staphylococci in our series) would be retested after 4 to 6 weeks to provide a definitive result, a modality that was not possible within the framework of the blinded prospective study.

In summary, this novel noninvasive approach based on multiplex antibody detection provides information not captured by common serological inflammation markers, hinting at the identification of several major PJI pathogens at the genus level. It might provide help in showing infections in patients without elevated ESR or CRP, allowing earlier management while preventing invasive and costly investigations. It might facilitate the interpretation of inconclusive bacteriological results (i.e., negative or single positive cultures) or P. acnes cultures. This approach should be evaluated and validated by further studies performed in different patient populations and countries, and its medicoeconomic benefit should be determined. The usefulness of this assay both at presentation of patients and as a follow-up tool should be further evaluated by longitudinal studies.

## Supplementary Material

Supplemental material
