# Supplementary material for: Multiplex Antibody Detection for Noninvasive Genus-Level Diagnosis of Prosthetic Joint Infection
Source: J Clin Microbiol. 2016 Mar 25;54(4):1065–73. doi: 10.1128/JCM.02885-15 (PMC4809921; doi:10.1128/JCM.02885-15)
Supplement: Supplemental material [file supp_54_4_1065__index.html]

Multiplex Antibody Detection for Noninvasive Genus-Level Diagnosis of Prosthetic Joint Infection — Supplemental material 

# Multiplex Antibody Detection for Noninvasive Genus-Level Diagnosis of Prosthetic Joint Infection

## Supplemental material

- Supplemental file 1 -

  Supplemental methods and Tables S1 (Microbial species involved in monomicrobial versus polymicrobial infections) and S2 (Performance of the multiplex immunoassay when undetermined results are excluded or are classified as either positive or negative)

  PDF, 170K
