# Supplementary material for: Multiplex Antibody Detection for Noninvasive Genus-Level Diagnosis of Prosthetic Joint Infection
Source: J Clin Microbiol. 2016 Mar 25;54(4):1065–73. doi: 10.1128/JCM.02885-15 (PMC4809921; doi:10.1128/JCM.02885-15)
Supplement: Supplemental material [file JCM.02885-15_zjm999094902so1.pdf]

Supplementary data

COMPARATIVE IMMUNOPROTEOMICS

Two-dimensional gel electrophoresis (2-DE) and immunoblot analyses were performed on the cell pellet and supernatant fractions obtained from planktonic bacterial cultures in liquid medium at 37°C (*Staphylococcu*s sp., 6-h aerobic culture in tryptic soy broth; *S. agalactiae*, 2-day anaerobic culture in Todd Hewitt broth; *P.acnes*, 14-day anaerobic culture in brain heart infusion supplemented with 0.5% (w/v) glucose). Bacterial proteins were separated by isoelectric focusing and run on a 12.5% polyacrylamide gel, blotted onto 0.45 µm nitrocellulose membranes, and probed with appropriate dilutions of 5 to 7 pooled serum samples from PJI cases or controls. Alkaline phosphatase-conjugated goat anti-human immunoglobulin G was used as a secondary antibody. Gels and blots were scanned with a GS-800 scanner (Bio-Rad) and saved as .jpeg files using Quantity One. Spots were analyzed with MGI PhotoSuite II (MGI SoftwareCorp., Richmond Hill, Canada). Proteins from "differential" spots (*i.e*, only present or more intense in PJI pools than in controls) were analyzed using MALDI-TOF/TOF mass spectrometry following in-gel tryptic digestion.

Supplementary Table 1. Microbial species involved in monomicrobial *versus* polymicrobial infections[a].

Microbial species	Number (%) of cases		
	All cases (n=167)[b]	Monomicrobial cases (n=143)	Polymicrobial cases (n=24)
Staphylococcus species			
S. aureus	58 (34.7)	45 (31.5)	13 (54.2)
S. epidermidis	39 (23.4)	30 (21)	9 (37.5)
S. lugdunensis	9 (5.4)	8 (5.6)	1 (4.2)
S. capitis	7 (4.2)	6 (4.2)	1 (4.2)
S. caprae	1 (0.6)	1 (0.7)	0
S. haemolyticus	1 (0.6)	0	1 (4.2)
S. hominis	1 (0.6)	1 (0.7)	0
S. xylosus	1 (0.6)	1 (0.7)	0
Streptococcus species			
S. agalactiae	8 (4.8)	6 (4.2)	2 (8.3)
S. bovis	1 (0.6)	1 (0.7)	0
S. dysgalactiae	1 (0.6)	1 (0.7)	0
S. oralis	2 (1.2)	1 (0.7)	1 (4.2)
S. pneumoniae	2 (1.2)	2 (1.4)	0
S. sanguinis	1 (0.6)	1 (0.7)	0
Propionibacterium species			
P. acnes	13 (7.8)	10 (7)	3 (12.5)
P. avidum	3 (1.8)	2 (1.4)	1 (4.2)
Other bacterial species			
Acinetobacter ursingii	1 (0.6)	0	1 (4.2)
Anaeroccus hydrogenalis	1 (0.6)	0	1 (4.2)
Corynebacterium striatum	3 (1.8)	1 (0.7)	2 (8.3)
Enterobacter cloacae	7 (4.2)	2 (1.4)	5 (20.8)
Enterococcus faecalis	13 (7.8)	11 (7.7)	2 (8.3)
Enterococcus faecium	2 (1.2)	2 (1.4)	0
Escherichia coli	6 (3.6)	4 (2.8)	2 (8.3)
Finegoldia magna	4 (2.4)	0	4 (16.7)
Granulicatella adjacens	1 (0.6)	0	1 (4.2)
Klebsiella pneumoniae	2 (1.2)	1 (0.7)	1 (4.2)

Morganella morganii	1 (0.6)	0	1 (4.2)
Peptoniphilus harei	2 (1.2)	0	2 (8.3)
Pseudomonas aeruginosa	5 (3)	3 (2.1)	2 (8.3)
Salmonella sp.	2 (1.2)	2 (1.4)	0
Fungal species			
Candida albicans	1 (0.6)	1 (0.7)	0

[a] Monomicrobial: only one microbiologically significant agent (at least one intraoperative sample positive in culture for virulent organisms or at least two intraoperative samples positive with the same organism); polymicrobial: at least two agents reaching microbiological significance criteria.

.

Supplementary Table 2. Performance of the multiplex immunoassay when undetermined results are excluded, or classified as either positive or negative.

**Organisms**	**Exclusion of undetermined results**		**Classification of undetermined results as positive**		**Classification of undetermined results as negative**	
	**Sensitivity**	**Specificity**	**Sensitivity**	**Specificity**	**Sensitivity**	**Specificity**
Target staphylococci	68/94<br>72.3% [62.7, 80.7][a]	213/264<br>80.7% [75.6, 85.1]	75/101<br>74.3% [65.1, 82.1]	213/279<br>76.3% [71.1, 81.1]	68/101<br>67.3% [57.7, 75.9]	228/279<br>81.7% [76.9, 85.9]
S. aureus	36/54<br>66.7% [53.4, 78.2]	-	40/58<br>69% [56.2, 79.8]	-	36/58<br>62.1% [49.1, 73.8]	-
S. epidermidis	26/35<br>74.3% [58, 86.7]	-	30/39<br>76.9% [61.9, 88.1]	-	26/39<br>66.7% [50.9, 80]	-
S. lugdunensis	9/9<br>100% [71.7, 100]	-	9/9<br>100% [71.7, 100]	-	9/9<br>100% [71.7, 100]	-
Other staphylococci	6/11<br>54.5% [25.9, 81]	-	6/11<br>54.5% [25.9, 81]	-	6/11<br>54.5% [25.9, 81]	-
S. agalactiae	6/8<br>75% [38.8, 95.6]	250/270<br>92.6% [89, 95.3]	6/8<br>75% [38.8, 95.6]	250/279<br>89.6% [85.6, 92.8]	6/8<br>75% [38.8, 95.6]	259/279<br>92.8% [89.3, 95.4]
Other streptococci	5/22<br>22.7% [8.8, 43.4]	-	5/22<br>22.7% [8.8, 43.4]	-	5/22<br>22.7% [8.8, 43.4]	-
P. acnes	5/13<br>38.5% [15.7, 65.9]	235/277<br>84.8% [80.2, 88.7]	5/13<br>38.5% [15.7, 65.9]	235/279<br>84.2% [79.6, 88.2]	5/13<br>38.5% [15.7, 65.9]	237/279<br>84.9% [80.4, 88.8]
Other *Propionibacterium*	2/3<br>66.7% [13.2, 98.3]	-	2/3<br>66.7% [13.2, 98.3]	-	2/3<br>66.7% [13.2, 98.3]	-

[a] 95% CI.

Number (%) of undetermined results: i) infected cases: target staphylococci: 7 (6.9%); *S. agalactiae*: 0; *P. acnes*: 0; ii) non-infected cases: target staphylococci: 14 (5%); *S. agalactiae*: 8 (2.9%); *P. acnes* : 1 (0.4%).
